# Novel technologies uncover novel ‘anti’-microbial peptides in *Hydra* shaping the species-specific microbiome

**DOI:** 10.1098/rstb.2023.0058

**Published:** 2024-05-06

**Authors:** Alexander Klimovich, Thomas C. G. Bosch

**Affiliations:** Zoological Institute, Christian-Albrechts University of Kiel, Am Botanischen Garten 1-9, Kiel 24118, Germany

**Keywords:** taxonomically restricted genes, machine learning, scRNA-seq, holobiont

## Abstract

The freshwater polyp *Hydra* uses an elaborate innate immune machinery to maintain its specific microbiome. Major components of this toolkit are conserved Toll-like receptor (TLR)-mediated immune pathways and species-specific antimicrobial peptides (AMPs). Our study harnesses advanced technologies, such as high-throughput sequencing and machine learning, to uncover a high complexity of the *Hydra*'s AMPs repertoire. Functional analysis reveals that these AMPs are specific against diverse members of the *Hydra* microbiome and expressed in a spatially controlled pattern. Notably, in the outer epithelial layer, AMPs are produced mainly in the neurons. The neuron-derived AMPs are secreted directly into the glycocalyx, the habitat for symbiotic bacteria, and display high selectivity and spatial restriction of expression. In the endodermal layer, in contrast, endodermal epithelial cells produce an abundance of different AMPs including members of the arminin and hydramacin families, while gland cells secrete kazal-type protease inhibitors. Since the endodermal layer lines the gastric cavity devoid of symbiotic bacteria, we assume that endodermally secreted AMPs protect the gastric cavity from intruding pathogens. In conclusion, *Hydra* employs a complex set of AMPs expressed in distinct tissue layers and cell types to combat pathogens and to maintain a stable spatially organized microbiome.

This article is part of the theme issue ‘Sculpting the microbiome: how host factors determine and respond to microbial colonization’.

## 1. Introduction: diversity and role of antimicrobial peptides in *Hydra*

Antimicrobial peptides (AMPs) are small cationic peptides that play a crucial role in the innate immune defence of a wide range of organisms from bacteria to humans [1,2]. These peptides exhibit broad-spectrum activity against various microorganisms, including bacteria, fungi, viruses and parasites.

**Figure 1.**
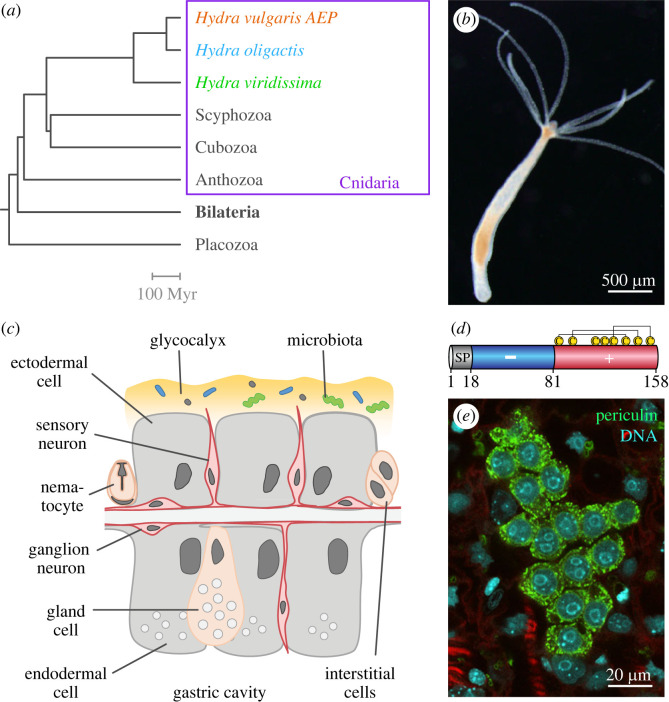
(*a*) Phylogenetic tree demonstrating the position of *Hydra*. High-quality genome datasets have become recently available for three *Hydra* species—*H. vulgaris* AEP, *H. oligactis* and *H. viridissima*. The divergence of the crown group *Hydra* took place about 193 Ma, and two species of brown hydras, *H. vulgaris* and *H. oligactis*, diverged over 100 Ma [3]. (*b*) A polyp of *H. vulgaris* AEP strain. It is composed of a tube-shaped body column, a basal disc attaching to a substratum, and an oral end with a hypostome and ring of tentacles. (*c*) The *Hydra* body is composed of the ectodermal and endodermal epithelial layers separated by the extracellular matrix. The outer surface of the ectoderm is covered by a glycocalyx that serves as a habitat for symbiotic bacteria. The endoderm lining the gastric cavity is free of glycocalyx and stable microbiota. Cells of the interstitial lineage, including the stem cells, nematocytes, gland cells and the neurons, are embedded within both epithelia. (*d*) Hydra-restricted periculin protein demonstrates key features of *Hydra* AMPs—small size, presence of a signal peptide (SP), bi-partite charge distribution and complex pattern of Cys-bridges. (*e*) Periculin is specifically expressed in the female gamete precursor cells of *Hydra*. Immunochemical detection of periculin 1a, DNA stained with TO-PRO3.

The freshwater polyp *Hydra*, a member of the phylogenetically ancient phylum Cnidaria (figure 1*a–c*), has long been used as a model organism for the study of the immune response evolution [4–6]. Major components of the *Hydra* immune toolkit are highly conserved immune pathways mediated by Toll-like receptors (TLR) [5,7] and nucleotide-binding and oligomerisation domain-like receptors (NLR) [8]. They are complemented by a rich repertoire of immune effector molecules—secreted AMPs. While the first AMP in *Hydra* was discovered using traditional biochemical approaches [9], the advance of molecular biology techniques fueled the identification of multiple novel AMPs, such as the arminins, periculins, kazal-like inhibitors and the neuron-derived antimicrobial peptide NDA-1 [10–13]. AMPs in *Hydra* share several common features: active AMPs are derived from larger precursors through a post-translational proteolytic cleavage of a signal peptide (figure 1*d*). Most AMPs are characterized by a clear bipartite structure with a strongly biased distribution of positively and negatively charged amino acids, and a complex cysteine pattern. Another notable property of *Hydra* AMPs is that they are typically encoded by a number of paralogous genes, hence they represent distinct gene families. Importantly, the phylogenetic analysis of AMP genes in *Hydra* uncovered that no homologues of these genes can be found in other animals, outside of the *Hydra* genus. Therefore, most AMPs of *Hydra* appear to be species-specific and, hence, represent so called taxonomically restricted genes (TRGs) or orphans [14]. This suggests that the taxonomically restricted AMPs have evolved relatively recently in the evolution of *Hydra* and specifically in response to the unique challenges faced by this animal.

Studies on the *Hydra* AMPs function provided evidence that mature secreted peptides possess a specific and often remarkably strong antibacterial activity, and are able to effectively inhibit growth of gram-positive and -negative bacteria *in vitro* [9,11,13,15,16]. These observations led to a hypothesis that AMPs protect the *Hydra* from foreign microbes. Later, it was recognized that, *in vivo*, they are equally important for maintaining the diversity of the species-specific bacterial community stably associated with *Hydra*, the *Hydra* microbiome [1,17]. This has been convincingly demonstrated in experiments where genetic knock-down of individual AMP genes or their families resulted in profound changes in the *Hydra* microbiome composition [11,12,18].

Thus far, AMP genes and their products have been identified and functionally characterized individually, and no systematic study attempted to integrate the findings on the entire suite of AMPs present in each *Hydra* species. To understand the evolutionary dynamics of *Hydra*-specific AMPs and their functional role in maintaining microbiome homeostasis, a comprehensive, whole-genome-scale survey of the AMPs repertoire and their expression in *Hydra* is needed.

Here, we demonstrate how novel technologies, including high-throughput transcriptome and genome sequencing and machine learning, provide insights into a high complexity of the *Hydra′*s AMP repertoire. Further, we uncover a shared feature of AMP genes genomic organization and common principles that govern the tissue and cell type-specific expression of these genes. Furthermore, we explore the evolutionary significance of these genes and their role in sculpturing the *Hydra*-specific microbiome. Finally, we outline a few open question and perspectives for further research on this enigmatic group of genes.

## 2. Insights from genomes: AMPs are encoded by fast evolving genes

The first AMPs discovered in *Hydra*, hydramacin and hydralysin, were initially identified through biochemical purification from *Hydra* tissue extracts [9,19]. Recent advancements in molecular biology techniques, such as expressed sequence tag analysis (EST) [9,10] and high-throughput transcriptome sequencing (RNAseq) [13,15], have greatly facilitated the systematic discovery of novel AMPs in *Hydra*. The utilization of these technologies has greatly expanded our understanding of the diversity and complexity of AMP families in *Hydra*. However, it remained unclear how complete was the repertoire of AMPs in each *Hydra* species, and whether all members of AMP families have been discovered. Recently, high-quality genome sequences became publicly available (see figure 1*a*) for two species of the ‘brown hydra’ phylogenetic group (*Hydra vulgaris* AEP and *Hydra oligactis*) [20], and one green hydra species (*Hydra viridissima*) [21], hence providing a glimpse into 200 Myr of evolutionary radiation within the *Hydra* crown group [3]. Additionally, a number of high-quality genomes of other hydrozoan cnidarians, scyphozoans and anthozoans became available [22–26]. Together, these resources allow accurate analysis of AMP genes and may provide novel insights into the role of AMPs in the biology of *Hydra*.

**Figure 2.**
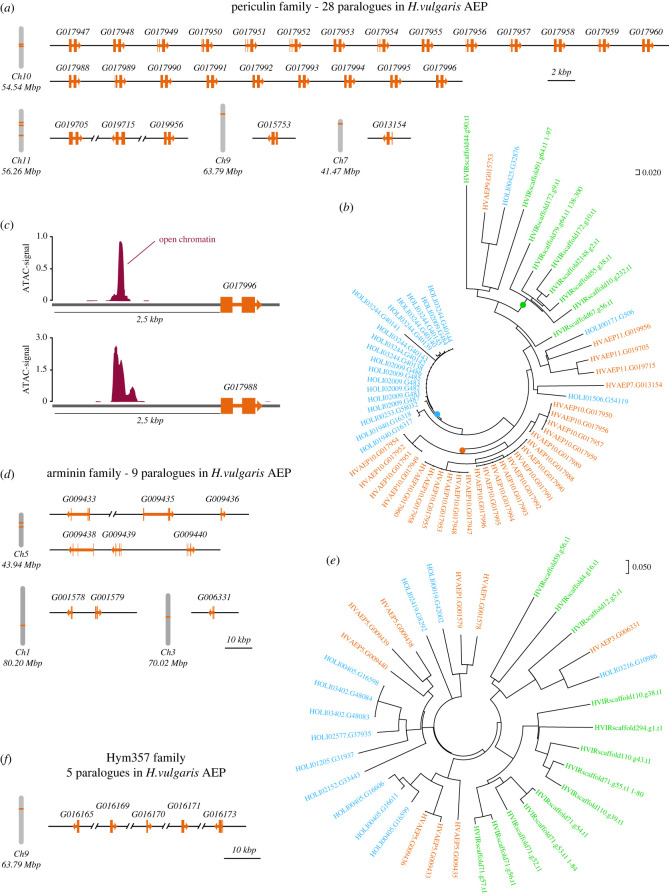
Complexity of AMP gene families in *Hydra* species. (*a*) Twenty-eight paralogues of the *periculin* AMP family are located on four chromosomes of *H. vulgaris* AEP, whereby 23 genes form one dense cluster on chromosome 10. (*b*) Phylogenetic analysis of *periculin* orthologues from three *Hydra* species. Genes are coloured according to species and numbers correspond to gene models: *H. vulgaris* AEP (HVAEP; orange), *H. oligactis* (HOLI; blue) and *H. viridissima* (HVIR; green). Not compressed bootstrapped tree is shown in electronic supplementary material, figure S3. (*c*) Chromatin accessibility analysis using ATAC-seq approach uncovers open chromatin regions within 2.5 kbp upstream from poorly expressed *periculin* genes, suggesting that these genes are not pseudogenes. Visualization based on data from Cazet *et al.* [20]. (*d*) Most *arminin* family paralogues are also present in one genomic locus on chromosome 5 in *H. vulgaris* AEP. (*e*) Phylogenetic analysis of *arminin* orthologues from three *Hydra* species. Not compressed bootstrapped tree is shown in electronic supplementary material, figure S4. (*f*) All five paralogues of Hym357 genes are found in one genomic cluster on chromosome 9 of *H. vulgaris* AEP. A complete list of accession numbers is presented in electronic supplementary material, table S1.

To uncover the complete repertoire of AMPs in *Hydra*, we first identified all paralogues of known AMP gene families in the genome of *Hydra vulgaris* strain AEP [20] (see electronic supplementary material text for details; electronic supplementary material, data). This strain is of particular interest, since it is the only one where functional gene manipulation by transgenesis is available [27,28]. In the *H. vulgaris* AEP genome, we discovered, to our surprise, a very high number of paralogues within each AMP family, often substantially higher than previously reported. For instance, we were able to identify at least 28 paralogues of *periculin* family genes (figure 2*a,b*; electronic supplementary material, table S1), in contrast to previously reported five *periculin* isoforms [13]. Although the nucleotide sequences of these 28 paralogues were clearly different (electronic supplementary material, figure S1), all these genes demonstrated similar exon-intron structure (figure 2*a*), and the amino acid sequences of peptides encoded by these genes were very remarkably similar (electronic supplementary material, figure S2). Most intriguingly, numerous *periculin* paralogues were found clustered in a few genomic loci (figure 2*a*). For instance, in *H. vulgaris* AEP, two clusters on chromosome 10 contained 14 and 9 *periculin* paralogues, and the other five paralogues were scattered among three other chromosomes. A very similar pattern was observed for other AMP families. We identified a total of nine *arminin* paralogues, seven genes of the Kazal-like family, and five genes encoding Hym357-like neuropeptides with antimicrobial activity (figure 2*d–f*; electronic supplementary material, table S1).

Taken together, these observations point to a substantial expansion of AMP gene families in *H. vulgaris* AEP. The genomic organization of the AMP gene clusters suggests that, during evolution, the peptide families were formed through several rounds of tandem gene duplications. This vast gene expansion appears particularly surprising given the relatively recent origin of the founder genes: for instance, *periculin* and *arminin* genes are strictly confined to the genus *Hydra* and, hence, their origin cannot date back longer that 200 Ma, and the duplication might have occurred much more recently. The mechanisms that may have contributed to the rapid evolution of the AMP gene complement in the recent history of the *Hydra* genus remain poorly understood.

To explore further the phylogenetic history of the duplicated AMP genes, we used available high-quality genomes of other *Hydra* species, as well as other cnidarians (see electronic supplementary material, text). This analysis of orthologues yielded three essential observations. First, the general trend of the presence of multiple paralogues has been confirmed. For instance, similar to *H. vulgaris* AEP, the genome of *H. oligactis* contained 21 paralogues of *periculin* family genes and 12 *arminin* orthologues (figure 2*b,e*; electronic supplementary material, figures S3 and S4; electronic supplementary material, table S1). These numerous paralogues of AMP genes were also clustered on the chromosomes of *H. oligactis* and *H. viridissima*, like in the *H. vulgaris* AEP genome (this is reflected in close numbers of the gene models from all three species; figure 2*b,e*; electronic supplementary material, table S1).

Second, the phylogenetic reconstruction uncovered that, in every *Hydra* species, AMP paralogues from each *Hydra* species tend to cluster together, forming species-specific clades (figure 2*b,e*; electronic supplementary material, figures S3 and S4). Typically, AMP genes from one species code for very similar or virtually identical proteins, distinct from AMPs from other species. For instance, 23 *periculin* paralogues in *H. vulgaris* AEP represent a solid cluster on the phylogenetic tree (figure 2*b*; electronic supplementary material, figure S3), and most likely have emerged from one ancestral sequence within *H. vulgaris* AEP. A set of 18 *periculin* paralogues in *H. oligactis* was formed independently (figure 2*b*; electronic supplementary material, figure S3).

This clear affinity of paralogues strongly supports their origination through a repeated and recent gene duplication within each *Hydra* species. In addition, in every *Hydra* species we uncovered individual representatives of AMP gene families that were clustering separately from closely related paralogues (figure 2*b,e*). These sequences represent, most likely, the ancestral, founder members of AMP families. Taken together, our cross-species analysis suggests that the ancestral state of the AMP gene complement was in fact very small, composed of three *periculin* and two *arminin* paralogues (figure 2*b,e*). These gene families underwent a major expansion later, upon radiation of *Hydra* species.

To our surprise, we were not able to uncover any *hydramacin* orthologues in *H. viridissima* using BLAST and hidden Markov model (HMM)-based searches (see electronic supplementary material, text), although two orthologues were confidently detected in the *H. oligactis* genome. Moreover, the synteny analysis (see electronic supplementary material, text) identified only a non-coding sequence in the syntenic *H. viridissima* chromosomal region where the *hydramacin* orthologue would be anticipated (electronic supplementary material, figure S5). This suggest that the ancestral arsenal of AMPs in the last common ancestor of green and brown hydras was very limited and did not include any hydramacin peptides, which evolved later, after the radiation of the crown *Hydra* group.

Finally, our screening for putative orthologues of AMP genes in the genomes of other cnidarians revealed no homologues even in closely related hydrozoans—*Hydractinia* and *Clytia*. These observations support the notion that AMPs are truly lineage-restricted genes confined to the *Hydra* genus. They evidently emerged about 200 Ma and diverged further following the radiation of *Hydra* species. The absence of any orthologues with at least partial similarity in animals outside of *Hydra* genus strongly suggests that the ancestral AMP genes have emerged *de novo* [29] from a non-coding sequence through one of multiple gene birth mechanism [30]. Although the origin of the founder AMP genes and the mechanisms of their further expansion in the *Hydra* lineage represent a substantial interest, they are beyond the scope of this study.

Similar to *Hydra*, the repertoire of AMPs in other animals and plants is dominated by lineage-specific genes. For instance, the cathelicidin peptide family is restricted to vertebrates [31–33], and diptericins are peptides confined to Diptera [34]. However, one AMP family, the defensins [35], appears to be omnipresent in the animal kingdom, in plants and fungi. Numerous *defensin* genes were *in silico* predicted from the genomes of Cnidaria as well [36–38] and few of them were empirically validated [39]. However, no members of the defensin family have been described in *Hydra* so far. We attempted to mine the genomes of three *Hydra* species for genes encoding putative defensins using BLAST and HMM-based approaches (see electronic supplementary material, text). To our surprise, we were not able to identify any genes in *Hydra* genomes coding for peptides with attributes of canonical defensin family members—mammalian defensins, arthropod defensins or protostome big defensins (electronic supplementary material, figure S6). Given that *defensin* orthologues are present in other cnidarians, placozoans and sponges, the most parsimonious explanations would be that the ancestral *defensin* genes were either lost in the *Hydra* lineage or evolved beyond recognition. We note that the *Hydra*-specific AMP hydramacin, in fact, shares some similarity with defensins (including the presence of six cystein residues), as previously suggested [9]. It is thus possible that hydramacin represents a far derived version of an ancestral defensin AMP.

Although we were not able to detect any *bona fide* defensins encoded in the *Hydra* genomes, our analysis uncovered a family of genes encoding secreted cysteine-rich peptides with partial similarity to defensins. Similar to defensins, these peptides possess six Cys residues, likely linked into three disulphide bonds, yet the spacing between these residues is clearly different from that characteristic for defensins (electronic supplementary material, figure S6, S7*a*). Additionally, these peptides are rich in tryptophan, and hence, we refer to them as *Hydra* cysteine/tryptophan-rich peptides, the HyCWR peptides. Intriguingly, the predicted HyCWR peptides demonstrated a strongly biased charge distribution, with the C-terminal portion being strongly positively charged, however no conventional cleavage site was found to separate these two portions (electronic supplementary material, figure S7*a*). We also note that the HyCWR genes represent a family of related genes, which comprises at least five orthologues in *H. vulgaris* AEP, seven in *H. oligactis* and one in *H. viridissima* (electronic supplementary material, figure S7*a*; electronic supplementary material, table S1), whereby several paralogues are typically located in the same genomic locus. Therefore, the HyCWR peptides in their structure and the genomic architecture of their genes follow similar trends described for AMPs in *Hydra*. However, we emphasize that it remains unclear whether the HyCWR peptides indeed display antimicrobial activity *in vitro* and *in vivo*. It is plausible that, in the absence of *bona fide* defensins, the non-related yet structurally similar HyCWR peptides take over their function. Taken together, a genome-wide mining for AMP sequences and cross-species comparison of AMP genes reveal a high complexity of AMP families in *Hydra* and suggest a complex gene family evolution within the *Hydra* genus.

## 3. Insights from scRNAseq—AMP genes are selectively expressed in certain cell types

Previous findings uncovered that most AMP genes are constitutively transcribed at a very high level. For instance, *arminin* mRNAs were reported to be more abundant that *β-actin* transcripts [12]. Similarly, *periculin* transcripts were among the most abundant transcripts in female polyps [13,40]. Additionally, AMP genes were reported to be expressed in certain tissue layers of *Hydra*. Most *arminin* paralogues, for instance, were expressed exclusively in the endodermal epithelial layer [12], while *periculin* transcripts were rather restricted to the female germline precursor cells within the interstitial cell lineage [13,40] (figure 1*e*). More recently, several AMP genes with neuron-restricted expression were discovered [11,16], but a comprehensive overview of the AMP cell-type specific expression pattern is still missing. Whole-genome expression atlases with single-cell resolution, which recently became available [16,41], uncovered a high diversity of cell types in *Hydra*. For instance, five types of ectodermal epithelial cells with specific transcriptional profiles and localization in the body were identified using scRNA sequencing. Even more surprisingly, up to 11 distinct spatially restricted neuronal cell types have been characterized [16,41,42]. Given this diversity of cell types, whole-genome expression atlases may provide a more comprehensive understanding of AMPs expression pattern and valuable insights into their function.

**Figure 3.**
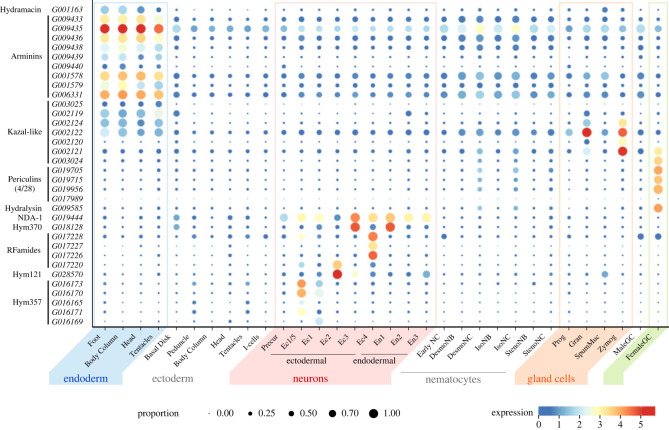
Expression of genes coding for AMPs across all cell types of *Hydra*. Visible are only the genes constitutively expressed in a *H. vulgaris* AEP polyp in homeostatic conditions, while inducible AMP genes expression is not illustrated. Note that only four out of 28 *periculin* paralogues are expressed at detectable levels. Visualization is based on data from Cazet *et al.* [20], gene expression is normalized and log-transformed.

Our mapping of AMP genes expression using the scRNA-seq atlas of *H. vulgaris* AEP [20,41] fully corroborated and expanded earlier observations (figure 3). Indeed, the *hydramacin*, all *arminin* and most *kazal-like* genes are expressed exclusively in the endodermal epithelial cells (figure 3; electronic supplementary material, figure S8). Moreover, several other *kazal-like* transcripts are expressed in the gland cells, also located strictly in the endodermal layer. The ectodermal epithelial cells, on the contrary, were generally devoid of any AMP gene transcripts (figure 3). Our preliminary observations suggest that the genes encoding HyCWR peptides might be the only group of AMPs expressed in the ectodermal cells (electronic supplementary material, figure S7*b,c*). The cells of the interstitial lineage localized in the ectodermal layer (figure 1*c*), however, do express a variety of AMP genes. First, female germline precursor cells produce transcripts encoding the hydralysin, several periculin and Kazal-like peptides (figure 3). Neurons localized in the ectodermal and endodermal layers (figure 1*c*) express distinct sets of dual function peptides, such as Hym370, Hym176, RFamides [11] and Hym121 [16]. Only one of these neuron-specific AMPs, NDA-1, is produced by both ectodermal and endodermal neurons.

The scRNA-seq data also provided insights into the spatial expression of AMP genes along the body axis of *Hydra*. AMP genes expressed in the endodermal epithelial cells do not show any expression bias and their transcripts are equally abundant in the polyp's foot, body column, head and tentacles (figure 3). Ectodermally expressed genes coding for putative HyCWR peptides show more distinct expression patterns, whereby one of them (G021955; electronic supplementary material, figure S7*b,c*), for instance, is strongly expressed in the basal disc, while another paralogue is not expressed in the foot at all (G0114589, electronic supplementary material, figure S7*b,c*). Expression of Kazal-like AMPs is confined to the upper body column, since zymogen and granular gland cells are abundant in the upper gastric region, but virtually absent from the polyp's foot and tentacles [41,43]. Intriguingly, since most of the neuronal populations are spatially restricted [16,41], the expression of neuron-derived AMP genes is also confined to a particular body compartment of *Hydra*. For instance, two RFamide precursor genes are expressed only in the hypostome and tentacles (population Ec4, figure 3), while Hym121 precursor is strictly present in the tentacles (neuronal population Ec2). Therefore, in each part of a polyp, a complex cocktail of AMPs is produced collectively by a variety of cell types.

The scRNA-seq datasets along with *in situ* hybridizations provide valuable insights into the expression of AMP genes on mRNA level. However, the localization of mature peptides translated from these mRNAs remains poorly investigated. Owing to the availability of specific antibodies, the localization of periculin peptides has been studied in most detail [13,40]. Mature periculins are produced in the female germline cells (figure 1*e*) located in the polyps ectoderm, are secreted and found on the outer surface of the epithelium. Even more intriguingly, periculins are also accumulated in the vesicles within the nurse cells, incorporated into an oocyte and released onto the embryo surface beneath the cuticle layer at early gastrulation stages [13]. Additionally, a fusion protein periculin-GFP expressed in the ectodermal epithelial cells recapitulates the vesicular accumulation and release of the peptide on the surface [13]. Similarly, with the help of specific antibodies, deposition of the neuronally expressed peptide NDA-1 into the glycocalyx of *Hydra* has been also demonstrated [11]. Therefore, the glycocalyx appears impregnated with diverse AMPs. Further proteome studies using mass spectrometry approaches, and particularly, spatial proteomics [44], should provide a more comprehensive view of the AMP localization in diverse cells, tissues and body compartments of *Hydra*.

Another evident observation emerging from the scRNA-seq data is that a substantial fraction of AMP genes is actually not expressed in homeostatic conditions. For instance, 24 out of 28 *periculin* paralogues have no evidence for transcription in the scRNAseq atlas, while several other AMP genes demonstrate a barely detectable expression in a small proportion of cells (figure 3). This is consistent with earlier observations of Franzenburg and co-authors [12], who reported expression of some *arminin* paralogues to be below detection level of microarray hybridization.

A plausible explanation for this observation might be that numerous paralogues of AMP genes actually represent pseudogenes. However, several lines of evidence speak against this assumption. First, all paralogues, including the non-expressed ones, display features of protein-coding genes, such as an open-reading frame with a defined transcription start site, a start and a stop codon. Second, the paralogues show very similar exon-intron structure (particularly evident in case of *periculins*, figure 2*a*). Third, the sequences of these paralogs do not overlap with coding sequences of other genes. Finally, ATAC-seq profiling of the accessible chromatin states [20] identifies distinct peaks about 2.5 kbp upstream from the coding sequences of AMP genes, even the ones that have no expression evidence (figure 2*f*). Such a pattern of ATAC-signal is characteristic for most *Hydra* promoters [20] and suggests that *cis*-regulatory elements upstream from the AMP genes are located in the open chromatin and are accessible for binding of transcription factors. In homeostatic conditions, although the genes appear silent, their promoters are primed, and the transcription of AMP genes can be effectively activated upon specific stimulus. Taken together, cumulative evidence clearly indicates that numerous poorly expressed *periculin* and *arminin* paralogues are true protein-coding genes, whose expression is silenced in homeostatic conditions (see also §6c).

## 4. Advances in artificial intelligence: AMPs can be predicted *ab initio*

**Figure 4.**
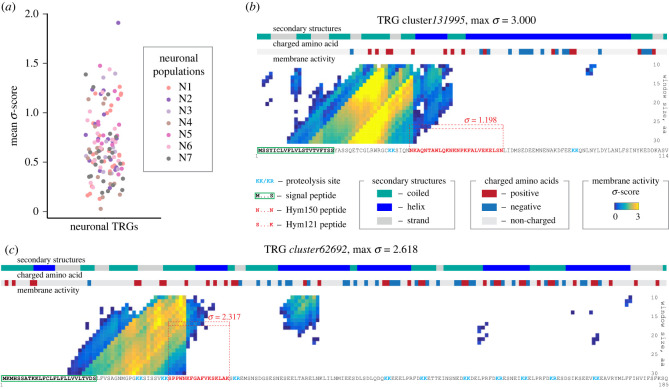
Machine learning algorithms allow for unbiased genome-wide prediction of putative AMPs. (*a*) Distribution of mean σ-score values for individual secreted peptides encoded by neuron-specific TRGs in seven neuronal subpopulations illustrates a high likelihood of containing active AMPs for the peptides. Data are from Klimovich *et al.* [16]. (*b*) *Hydra*-specific TRG *cluster131995* expressed exclusively in endodermal neurons N5 encodes a putative hitherto uncharacterized AMP. Moving-window protein scan prediction map with residue charge and secondary structure annotations. The heat map reflects the peptide's probability (σ-score) of being membrane active as predicted by the MLA [57]. High σ-scores (yellow) suggest that *cluster131995* peptide codes for a potent AMP. N-terminal signal peptide, putative proteolysis sites and a sequence identical to a previously discovered peptide Hym150 [58] are found within the cluster131995 peptide, providing evidence that a precursor *cluster131995* is processed and gives rise to a secreted active AMP. (*c*) The predicted profile of the peptide encoded in *cluster131995* resembles that of the TRG *cluster62692*, which has been previously demonstrated to contain a highly potent neuron-derived AMP Hym121 in *Hydra* [16].

Until recently, identification of AMPs in diverse organisms relied mainly on homology-based screenings using known peptides as a ‘bait’. Numerous AMP databases, such as the APD3, DBAASP, GRAMPA and InverPep [45–48], contain thousands of identified AMPs from animals and fungi, plants and microorganisms, provide a rich source of reference peptides for similarity searches and offer diverse build-in tools to perform such screenings. However, the homology-based approach has clear limitations, particularly given that, across the animal tree, AMPs are typically encoded by species-restricted genes [49]. Recent advances in artificial intelligence (AI), including the deep- and machine learning algorithms, provide a new opportunity of systematic *ab initio* discovery of novel AMPs [50–56]. Similar to BLAST-based homology searches, AI tools are dependent on rich datasets of known AMPs. However, in contrast to other approaches, AI predictive tools do not rely specifically on the amino acid sequence of AMPs. Instead, they identify essential physicochemical determinants of AMP functionality in the known AMPs present in the training dataset (so-called structure–function relations, which often are much more complex than simply a presence of a given amino acid in a certain position) and screen the target dataset to uncover proteins with similar structure–function correlations and rank them by likelihood of being *bona fide* AMPs. We have previously used one of these machine-learning algorithms (MLA) [57] to identify putative transcripts encoding α-helical AMPs among genes specifically expressed in *Hydra* neurons [16]. This approach turned out to be very effective and resulted in identification of dozens of putative neuronally expressed secreted AMPs encoded in Cnidaria-specific TRGs (figure 4*a*). These hitherto uncharacterized peptides, such as the product of a TRG *cluster131995* (figure 4*b*) demonstrate a very distinct pattern of charge and secondary structure distribution as well as strong predicted membrane activity. One of these genes, a *Hydra*-specific TRG *cluster62692*, was predicted to encode a precursor of a secreted short peptide with strong antimicrobial activity. Our minimal growth inhibitory concentration (MIC) assays confirmed that the active peptide Hym121 encoded within *cluster62692* was indeed a neuron-derived AMP highly potent against gram-positive and negative bacteria [16]. Hence, our functional analysis confirmed the accuracy of the MLA prediction. Intriguingly, a similar approach and the same MLA were used to identify a novel antimicrobial factor PACAP in the mammalian brain [59]. This dual-function neuropeptide known to regulate neurodevelopment, emotion and stress responses has been recently demonstrated to function as an AMP. Together, these observations demonstrate the power of AI tools in discovering novel functionally relevant AMPs. They also provide additional evidence, from the evolutionary perspective, for the structural similarity and functional reciprocity of AMPs and neuropeptides [60–62].

In our previous study, we focused on discovering putative AMPs exclusively expressed in neurons of *H. vulgaris* AEP. A high computational demand of the MLA precluded us from a deeper and more extensive analysis of AMP coding genes in *Hydra*. Nowadays, with the complete genomes for several *Hydra* species available and dramatically increased computational power, a whole-genome survey of AMPs encoded in *Hydra* genomes is feasible. It will be instrumental in uncovering novel, previously uncharacterized and very likely species-restricted AMP.

## 5. Lessons from the *Hydra* holobiont

### (a) Expansion of AMP families in the phylogenetically younger *Hydra* species

As any other animal, each *Hydra* species forms a stable association with a specific multispecies bacterial community and hence functions as a metaorganism [12,63]. Understanding the mechanisms and molecular interactions involved in long-term maintenance of the metaorganism homeostasis remains a major challenge. Since AMPs are key factors regulating bacterial colonization, it is imperative to consider our findings on the AMP complexity in *Hydra* in the holobiont framework.

**Figure 5.**
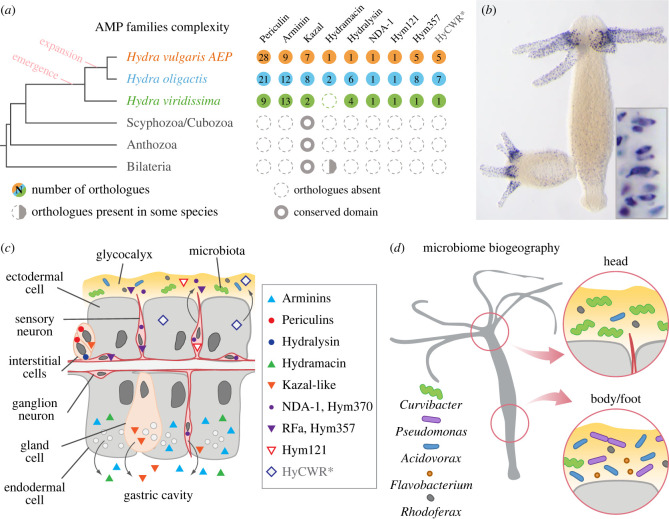
Complex species-specific and spatially restricted cocktails of AMPs sculpt the microbiome of *Hydra*. (*a*) Overview of the AMP gene family complexity in *Hydra* species. Note that *H. viridissima* possesses generally less AMP genes compared to *H. oligactis* and *H. vulgaris* AEP. Most AMP families are restricted to *Hydra* and only in a few cases can proteins with similar domains be detected in other cnidarians and/or bilaterian animals. (*b*) *Hydra*-specific AMP Hym121 is expressed in a distinct population of sensory neurons (N7) confined to the tentacles of *Hydra*, where it creates a selective microenvironment for specific members of the microbiome. *In situ* hybridization reveals the presence of Hym121 mRNAs. (*c*) Tissue and cell type-specific expression pattern of *Hydra*'s AMPs. While numerous AMPs are secreted into the gastric cavity by the endodermal layer, ectodermal epithelial cells may produce only few AMPs. This is particularly surprising given that the ectoderm is facing the environment. The AMPs produced by the neurons in the ectoderm are secreted directly into the glycocalyx, the habitat for symbiotic bacteria. (*d*) The sharp spatially controlled expression patterns of AMPs control the spatial organization of the *Hydra* microbiome—its biogeography. Asterisk, the antimicrobial activity of HyCWR peptides and their role in the *Hydra* holobiont remain to be validated.

First, our observations clearly indicate that the majority of *Hydra* AMPs are encoded in *Hydra*-restricted genes. The forces that propelled the emergence of these TRGs at the root *Hydra* (figure 5*a*) radiation about 190 Ma [3] remain unclear. It is, however, plausible that the transition of a *Hydra* ancestor from the marine into the freshwater habitat has exposed the host to a totally new microbial environment. Additionally, in the new freshwater, low ion-strength environment, some ancient AMPs might become inefficient (e.g. defensins are generally known to be highly effective in a saltwater environment and tend to expand in the context of marine habitats [64]). Together, these factors might have fuelled an elaboration of a new molecular language for communication between the host and the microbes.

Our genome-wide survey of AMP-encoding genes in *Hydra* uncovered a high complexity of lineage-restricted AMP families (figure 5*a*). While comparing different *Hydra* species, one interesting tendency became obvious: the size of AMP families was generally larger in the representatives of the ‘brown hydra’ group compared to the green hydra *H. viridissima* (figures 2 and 5). For instance, only eight *periculin* genes and a single *Hym357* orthologue were found in the *H. viridissima* genome, and *hydramacin* appears to be missing in this species. This trend suggests that a major expansion of AMP gene families has occurred after the segregation of the ‘green’ and ‘brown’ hydra groups, which took place about 193 Ma [3]. This phylogenetic radiation coincides with a major change in the *Hydra* biology—the loss of its algal photosymbiont *Chlorella*. Given the tight metabolic co-dependence between *H. viridissima* and its endosymbiont *Chlorella* [65], such a transition must have been reflected in the entire holobiont biology and most likely had an impact on the relation with the extracellularly located microbiota. It is plausible that, upon the partner switch, certain function(s) previously allocated to the photosymbiont might have been re-allocated (outsourced) to the bacterial symbionts. This, in turn, necessitated a more elaborate system of control exerted by *Hydra* on its microbiome in the form of AMPs. This scenario is supported by the observation that symbiotic *H. viridissima* harbour a distinct microbiome clearly different from that of aposymbiotic (algae-free) polyps [66]. We also note that colonization of *H. viridissima* with *Chlorella* algae is associated with an up-regulation of multiple *Hydra*-restricted TRGs [65], which remain uncharacterized, but some of them might code for putative AMPs.

These observations prompt a hypothesis that the loss of a photosynthetic endosymbiont might be associated with the increasing role of the extracellular bacterial microbiome, which demands a more sophisticated control via complex AMP cocktails. To test the hypothesis whether the bi- or tripartite holobionts architecture is reflected in the complexity and evolutionary history of their AMP genes repertoire, a comprehensive analysis across members of the Cnidaria phylum is imperative. While virtually all Anthozoa species form stable association with intracellular photobionts and species-specific bacterial communities colonizing the surface mucus layer, the gastrovascular system, and the skeleton [67], members of other Cnidaria classes, such as Scyphozoa and Hydrozoa, rarely harbour photobionts. *H. viridissima* and *Cassiopea xamachana* are, in fact, rather exceptions among the hydrozoans [68]. Although some recent studies attempt to create a comprehensive survey of AMPs in Cnidaria [36], their focus remains bound to exclusively conserved gene families. Implementation of novel highly automatized algorithms for AMP detection and annotation, such as the MLAs, promises a major progress in understanding the link between AMP repertoire and holobiont architecture in Cnidaria.

In this context, it is particularly interesting to compare the diversification of AMPs to the evolutionary history of other immune genes in *Hydra* and other symbiotic and non-symbiotic Cnidaria. The diversity of TLR genes in *Hydra* is very low. In fact, only a single functional TLR is assembled from the products of two genes—*hyLRR* and *hyTRR* [5,7]. Genes coding for putative NOD-like receptors, on the contrary, have undergone expansion in *Hydra* indeed [8]. Intriguingly, the broadest repertoire of genes encoding NACHT- and NB-ARC-domain containing NOD-like receptors (over 260 in total) is observed in the green *H. viridissima* [21]. The arsenal of NLRs in the brown *H. vulgaris* is substantially smaller (about 89–101 genes). Hence, we observe here an inverse trend, compared to the AMP families—expansion of a gene family in the context of algal symbiosis and contraction in algae-free hydras. Remarkably, this trend is also evident on the scale of the phylum Cnidaria: symbiotic cnidarians, like the anemone *Acropora*, possess over 400 NLR genes, while a symbiont-free jellyfish *Morbakka* has only 24 genes, and *Nematostella* has only six genes for NLRs. Similarly, TIR-domain-containing proteins are substantially more abundant in *H. viridissima* (49) and *Acropora* (49) compared to symbiont-free *H. vulgaris* (11) and *Nematostella* (17) [21]. Therefore, the evolutionary development of symbiosis with algae by certain cnidarians likely required expansion and greater sophistication of genes encoding innate immunity pathway genes, critical for recognition and maintenance of symbiotic organisms in cnidarian tissues. The loss of photosynthetic symbionts resulted in contraction of the receptor-encoding gene families and expansion of the families encoding the effector molecules for communication with the prokaryotic partners—the AMPs.

In sum, the emergence and ‘recent’ elaboration of the AMP repertoire in the brown *Hydra* might be a signal of a change in the holobiont complexity and biology. The complexity of AMP families in diverse *Hydra* species, hence, represents a genomic footprint of a co-evolution between the host, similar to other species (e.g. the fly [69]) and its microbiome and reflects the species' adaptations to their unique microbial environments.

### (b) AMPs shape the spatiotemporal structure of the *Hydra* microbiome

For several decades, it has been accepted that AMPs, as ‘killers’ and hence often referred to as host-defence peptides (HDP; [70]), protect an animal from noxious microorganisms. More recently, as stated above, we start appreciating a broader role of AMPs in shaping the commensal microbiome [71]. The *Hydra* host imposes strong selective forces on its microbiome via section of diverse AMPs [72] and thereby maintains species-specific microbiota communities over extended periods [12,63,73]. Our observations expand this view and add a spatial dimension to these host–microbiome interactions. We provide evidence that AMPs in *Hydra* are expressed in a tightly regulated spatially controlled manner (figure 5*b*). A plethora of AMPs are expressed throughout the endoderm of *Hydra* along the entire body (figure 3). These peptides, most likely, keep the gastric cavity of a polyp essentially sterile and protect *Hydra* from pathogens. In the ectodermal layer, AMPs expressed mostly in distinct spatially restricted neuronal populations are, hence, confined to certain body domains (figures 3 and 5). This suggests that spatially confined AMP cocktails are secreted into the glycocalyx of *Hydra* and generate a complex chemical landscape on the polyp's surface. These distinct microhabitats shape locally the microbiome of *Hydra*. They not only regulate the density of the bacterial communities a healthy polyp harbours (so called carrying capacity; [74]) but also control the composition of these communities. As a result, certain species of bacteria, such as *Pseudomonas*, *Flavobacterium* and *Acidovorax*, are confined to the lower part of the *Hydra* polyp and virtually absent from the hypostome [75]. On the contrary, other members of the microbiome such as *Curvibacter* [11,76], are found more abundantly on the polyp's head and tentacles. Our analysis of AMP genes expression uncovered their strict spatially restricted production and suggested their contribution to the specific regionalization of the microbiome (figure 5*b–d*).

We have mechanistically proven the role of *Hydra′*s AMPS in shaping the microbial biogeography by genetically modifying the expression pattern of a nerve cell-specific AMP, NDA-1 [11]. Using a knockdown approach, we observed that the absence of NDA-1 peptides results in both a shift in the composition of the microbiome and a perturbation of the microbial biogeography [11].

## 6. Perspectives and open questions

Our bioinformatic analysis uncovered a remarkable expansion of AMPs families encoded in *Hydra*-specific TRGs. However, to fully understand the evolution of the AMP gene complement and implications of this complexity for the *Hydra* holobiont, further systematic studies are needed.

### (a) Puzzling redundancy of AMP genes

Our analysis uncovered high evolutionary dynamics of AMP families in *Hydra*. Generally, duplication of species-specific AMP genes or their loss through pseudogenization are not uncommon in the animal kingdom [49,77,78], and most animals indeed possess a broad array of AMPs. The clustered genomic organization of AMP genes has also been recognized as characteristic for numerous AMP families across animal species [79–83]. However, we find it truly puzzling that numerous paralogues of AMP genes in *Hydra* though having slightly different nucleotide sequences, code for identical precursor polypeptides and, hence, give rise to identical active peptides. This is particularly evident in the case of the periculin family (figure 2*b*; electronic supplementary material, figure S2). The biological relevance of this apparent redundancy as well as the evolutionary mechanisms that lead to it remain unclear. A deep analysis of the paralogues' nucleotide sequences, such as the dN/dS estimation, may reveal sign of negative or positive selection. Additionally, comparison of gene complement and genomic organization between polyps from different geographically isolated populations of the same species might be informative. One can anticipate that such survey may even uncover single amino acid polymorphisms (similar, for instance, to the functionally crucial polymorphism S69R in diptericin A sequences [69]) or copy number variation in AMP genes within different *Hydra* clones. The current state of accuracy in genome sequencing and assembly allows detecting such genomic events.

In sum, the genome-wide survey of AMP repertoire in *Hydra* provides evidence for an expansion of AMP gene families. Together with observations on other invertebrate animals, plants and fungi [64,78,84,85], these findings support the view that elaboration of the AMP arsenal through novel family emergence, gene duplication and diversitifcation is a common, universal principle in AMP genes evolution.

### (b) Uncovering further AMP families in *Hydra*

Our analysis was focused on a detailed analysis of previously identified AMP families in *Hydra*. Beyond that, we demonstrate how additional, novel tools allow discovering novel members of known families or even new families. For instance, using a hidden Markov model-based approach, we uncovered a novel family of putative secreted AMPs—the HyCWR family. Novel AI-based tools also allow unbiased genome-wide screening and *ab initio* detection of AMPs. Our preliminary analysis suggests that dozens of novel, previously not characterized AMPs and their families are still hidden in the genome unrecognized (figure 4*a*). This hypothesis is supported by our finding that clusters of tandemly repeated *Hydra*-specific TRGs, architecturally similar to, for instance, the *periculin* cluster (figure 2*a*), are scattered through the *Hydra* genome. For example, a dense cluster of over 30 relatively short collinear uncharacterized genes with no homologues outside *Hydra* (*G009076 – G009116*) can be found on chromosome 5 of *H. vulgaris* AEP.

Testing the hypothesis whether this plethora of genes encode novel AMPs and characterizing them represent a major analytical challenge. However, this analysis may be streamlined by applying improved AI tools. In our previous efforts, we trained the MLA using a dataset of predominantly human AMPs [57]. Therefore, our analysis had a certain bias and likely, favoured identifying AMPs with features common to those of Bilateria. However, the dynamic nature of MLA allows re-training them on additional or expanded datasets. Addition of already discovered and functionally validated AMPs from *Hydra* into the training dataset may substantially increase the accuracy of the MLAs. Moreover, the rapidly evolving tools for three-dimensional protein modelling, such as the AlphaFold and similar template-independent tools [86–88], offer an opportunity to predict with high confidence the folding of peptides and, hence, may greatly streamline the *in silico* analysis of putative AMPs and facilitate selection of candidates for testing *in vivo* and *in vitro*. We particularly emphasize that testing the function of candidate AMPs remains a major bottleneck. Not all peptides can be synthetized effectively in their active form and tested *in vitro*, and recombinant expression may be also challenging due to toxicity for cells. Finally, the *in vivo* studies of AMPs by manipulating the genes in the host though transgenesis are very laborious and require smart selection of candidates. AI algorithms represent an excellent tool for making an ‘educated guess’ and selecting candidates for in-depth validation.

The majority of AMPs in *Hydra* are encoded in *Hydra*-restricted TRGs (figure 5*a*), yet the *hydramacin* family is an exception. It appears to be confined to the brown *Hydra* group, since no orthologues were found in *H. viridissima* (electronic supplementary material, figure S5). This suggests that *hydramacin* either has emerged after the split of brown and green hydras, or has been lost in *H. viridissima*. The latter appears more plausible, since genes coding for proteins similar to hydramacin were found in several bilaterian species, such as leeches and molluscs [5,9,89,90]. Our synteny analysis (electronic supplementary material, figure S5), which indicates that the entire locus containing the *hydramacin* gene might have been lost in the green hydra lineage, provides an additional support for this hypothesis. Hence, the most parsimonious explanation of the mosaic *hydramacin* distribution on the phylogenetic tree is that the hydramacin family is ancient and likely common for all Eumetazoa, but its members have been either lost in some lineages or evolved beyond the level of detection. This gene loss might be not the only example of reduction in AMP repertoire in *Hydra*. For instance, our analysis provided no evidence for the presence of canonical defensins in *Hydra*. This appears surprising given a broad phylogenetic distribution of these peptides. However, partial or complete absence of some AMP families has been described in vertebrate and invertebrate species [34,77,91–93], supporting the high evolutionary dynamics of AMP families. To resolve the paradoxical absence of some AMP families in *Hydra* and identify the factors that might have caused this gene loss, a deep cross-species and, possibly, cross-isolate comparison of the genomic organization (exon-intron structure, synteny) are needed along with a survey of the microbiomes and the ecology of these species and isolates. Extensive implementation of AI tools may facilitate genome-wide discovery and comparison of AMP repertoires.

### (c) Uncovering expression of ‘silent’ AMP genes

Although many AMP genes discovered in *Hydra* are characterized by a constitutive expression, a substantial fraction of AMP genes appears not to be expressed in homeostatic conditions (figure 3; electronic supplementary material, figure S7). This suggests that AMP expression is under a tight developmental as well as environmental control. In fact, some *periculin* genes are developmentally regulated and expressed at particularly high level in mature oocytes. As maternal antimicrobial peptides, they control bacterial colonization of the *Hydra* embryos [13]. Absence of expression in adult polyps also indicates that some AMPs might be inducible, and their expression is triggered upon a specific signal, such as encounter of a bacterial species or metabolite. Indeed, earlier observations provide strong evidence that expression of some *hydramacin*, *arminin* and *periculin* paralogues can be up-regulated in the presence of diverse bacterial products (LPS, flagellin) or danger signals (dsRNA) [5]. Moreover, interference with the upstream signalling pathways [18], and tissue manipulations such as amputation-induced regeneration [94] and elimination of neurons [95,96], also result in modulation of AMP gene expression. This may cause a concomitant enhanced antimicrobial activity of the tissue [97]. However, it remains unclear, whether the transcription of already expressed paralogues is elevated, or additional previously silent AMP genes (figures 2*c* and 3) are turned on. A particularly exciting possibility is that ectodermal cells that do not produce any AMPs in homeostatic condition (figures 3 and 5*c*), start expressing certain AMP genes following developmental or environmental signals.

### (d) For AMPs the name no longer fits the function

As outlined in detail in another paper in this issue [98], from the beginning of animal (and plant) evolution, AMPs serve a crucial role in regulating the composition of the microbiome [1]. These findings make it quite clear that AMPs do much more than just kill pathogens. They play a ‘silent’ role in plant, animal and human health by permitting coexistence with environmental and symbiotic microbes, shaping the microbiome according to the susceptibility to particular AMPs, contributing to the spatial organization of the microbiota. Instead of being ‘anti’-microbial, one could just as well speak of ‘pro’-microbial peptides. The function of AMPs goes far beyond just killing bacteria. It is generally accepted that AMPs inhibit growth of microbes, through interfering with a diversity of cellular function in bacterial cells [2]. However, they can also interfere with the microbes physiology in plethora of other ways. Accumulating evidence indicates that AMPs may modulate formation of biofilms and swarming behaviour of microbes [99], or act as immunomodulators [100]. AMPs produced by *Hydra* may appear to display similar multifunctionality. Most of them do demonstrate strong growth-inhibiting activity in minimal inhibitory concentration (MIC) assays [9–11]. However, we noticed that some peptides have milder effects on target bacteria and rather change their physiology. For instance, that the Hym121 peptide effectively inhibits growth of *Curvibacter* and *Acidovorax*, but does not kill *Bacillus megaterium* and only alters its colony morphology, likely by reducing cells motility [16]. Therefore, this AMP actually acts as a signalling molecule (somehow reminiscent of the signalling role of microbe-derived antibiotics [101]). Similar observations remain very scarce, and no systematic survey of non-conventional roles of *Hydra* AMPs has been performed. Since MIC assays have been the main tools to test AMPs' activity and infer function, behaviour-modulating aspects of AMP activity have escaped detection so far. We emphasize the urgent need to develop and implement novel methods, such as motility assays and microcosm setups [74], to gain a comprehesive view of diverse AMP roles in animals. This thinking may also shape the development of *in silico* tools, such as activity predictors and AI-based algorithms (in line with the current efforts [56,102], whose logic has been mainly built around the membrane disruptive and bacteria killing properties. These developments may also fuel discovery and a guided design of novel antibiotics [56,103–105].

In sum, our survey of AMPs in *Hydra* uncovered a fascinating diversity and complex role of these TRGs in *Hydra* biology. It is generally accepted that emergence of novel, taxon-restricted genes may promote emergence of novel traits allowing access to a new environment. As demonstrated here, families of AMPs appear to represent an attractive system for experimentally dissecting the link between gene emergence and expansion, and a (meta)organism's phenotype and its adaptation to the environment. *Hydra* offers a unique experimental platform for testing how the host sculpts its microbiome, and the microbiome shapes the genome of its host. Hence, the studies on *Hydra* provide an evolutionary informed perspective onto the principles governing the intricate host–microbiome interactions and the molecular mechanisms behind them [17,106–108]. They enrich our understanding of the critical factors maintaining the metaorganism homeostasis and health across the animal kingdom.

## Data Availability

All data and references, such as Genbank accessions, are included in the manuscript and the electronic supplementary material. The sequence datasets supporting this article have been uploaded as part of the electronic supplementary material, table S1 and Data S1 and S2. Supplementary material is available online [109].
